# Managing Thoracic Aortic Aneurysm in Patients with Bicuspid Aortic Valve Based on Aortic Root-Involvement

**DOI:** 10.3389/fphys.2017.00397

**Published:** 2017-06-13

**Authors:** Elizabeth Norton, Bo Yang

**Affiliations:** ^1^Department of Internal Medicine, Michigan MedicineAnn Arbor, MI, United States; ^2^Department of Cardiac Surgery, Michigan MedicineAnn Arbor, MI, United States

**Keywords:** aortic valve, aortic valve stenosis, aortic valve insufficiency, aortic aneurysm, aortic dissection, bicuspid aortic valve

## Abstract

Bicuspid aortic valve (BAV) can be both sporadic and hereditary, is phenotypically variable, and genetically heterogeneous. The clinical presentation of BAV is diverse and commonly associated with a high prevalence of valvular dysfunction producing altered hemodynamics and aortic abnormalities (e.g., aneurysm and dissection). The thoracic aortic aneurysm (TAA) in BAV frequently involves the proximal aorta, including the aortic root, ascending aorta, and aortic arch, but spares the aorta distal to the aortic arch. While the ascending aortic aneurysm might be affected by both aortopathy and hemodynamics, the aortic root aneurysm is considered to be more of a consequence of aortopathy rather than hemodynamics, especially in younger patients. The management of aortic aneurysm in BAV has been very controversial because the molecular mechanism is unknown. Increasing evidence points toward the BAV root phenotype [aortic root dilation with aortic insufficiency (AI)] as having a higher risk of catastrophic aortic complications. We propose more aggressive surgical approaches toward the BAV with root phenotype.

## Introduction

Bicuspid aortic valve (BAV) affects 1–2% of the general population (Martin et al., 2007). Diagnosis of many patients born with BAV does not happen until adulthood; however, up to 50–70% of patients with BAV experience some form of complication such as valvular insufficiency or aortic aneurysm either as children or later in life. The underlying cause of the incorrect formation of the aortic valve remains relatively uncertain; however, evidence suggests that BAV is a genetic disorder. BAV exhibits an increased prevalence in first-degree relatives of affected individuals (Martin et al., 2007). The familial clustering suggests an autosomal dominant pattern of inheritance with reduced penetrance (Martin et al., 2007). There are both single and multiplex affected families, which indicates there may be multiple means of inheritance for BAV (Martin et al., 2007). Chromosomal regions of interest for BAV include 18q, 5q, and 13q (Martin et al., 2007). Despite these probable chromosomal regions, approximately 150 genes are encoded amongst the three novel loci (Martin et al., 2007).

Despite the establishment of the heritability of BAV, the genes yielding the pathology of the aortic valve remain largely undetermined. BAV consists of a variety of morphologies and depending on which cusps are fused, blood flow patterns are impacted, which can affect the aorta in various ways. The most prominent fusion is observed between the left and right coronary cusps, followed by the right and non-coronary cusps, and very few between the left and non-coronary cusps (Bissel et al., 2013). The Sievers classification organizes BAVs based on the different subtypes; AP, Lat, R-L, R-N, N-L, and L-R/R-N (Sievers and Schmidtke, 2007).

BAVs are associated with many clinically serious abnormalities, including aortic valve insufficiency and stenosis as well as aortic dilation and dissection. The management of thoracic aortic aneurysm (TAA) in BAV has been very controversial. The ACC/AHA guidelines have been changed back and forth in the past years regarding recommendation of surgical resection of TAA based on the size of aneurysm, between 5 and 5.5 cm. BAV/TAA has been treated in a uniform manner, despite the heterogeneity of the disease. We believe BAV/TAA should be treated based on phenotype of aortic root involvement. We provide an overview of the heterogeneity of BAV, and the associated complications to improve treatment. BAV with root phenotype TAA [aortic root dilation and aortic insufficiency (AI)] should be treated more aggressively surgically; and less aggressively if the aortic root is not involved. Currently BAV/TAA is treated with a blanket approach, but additional research could lead to more phenotypic-specific guidelines to improve patient outcomes.

## Materials and methods

We performed a search of the PubMed database for articles on BAV from inception to March 2017. Articles were limited to those written in English. Additionally, references from key articles were manually searched in a backward cumulative fashion for additional articles.

## Results

### Genetics

While candidate genes have been identified, *NOTCH1, NKX2.5*, and *GATA5* are the best supported although further replication is warranted. Mutations in *NOTCH1* function both in BAV and calcific aortic disease. *NOTCH1* functions in both familial and sporadic BAV and is crucial during cardiac valve formation that promotes epithelial–mesenchymal transition (Kostina et al., 2016). For example, disruption of Notch signaling in transgenic mice is correlated with faulty neural crest cells patterning as well as unequal aortic valve leaflets with bicuspid-like morphology and aortic arch abnormalities characterized by disorganized aortic wall histology with dispersed vascular smooth muscle cells (Broberg and Therrien, 2015). While many studies have reported a linkage of BAV to *NOTCH1, NOTCH1* mutations do not function in BAV in all instances. In fact, the *NOTCH1* gene mutation may only be associated with a very small portion of patients with BAV. *NKX2.5*, a homeobox-containing transcription factor, is required for cardiogenic differentiation across species (Qu et al., 2014). A study that detected a novel heterozygous sequence variation found that the mutation was present in all affected family members with BAV but absent from the unaffected family members (Qu et al., 2014). *GATA5* plays an essential role in cardiac morphogenesis and aortic valve development (Nemer et al., 1999). Due to its high expression in endocardial cushions of both the outflow tract and the atrioventricular canal, *GATA5* became a candidate gene for congenital heart diseases, more specifically BAV, and mutations in *GATA5* have been associated with an increased susceptibility to BAV, but the specific detailed molecular mechanism needs to be further researched. Through a GWAS study, we also identified a coding variant of GATA4 and a non-coding variant which is 150 kb away from GATA4 are associated with BAV (Yang et al., 2017). Despite the awareness of candidate genes for the heritability of BAV, the specific genetic basis underlying BAV remains largely unknown. The involvement of many genes adds to the heterogeneity of the population of patients with BAV. In addition to the genetic source leading to the formation of BAV, the complications of BAV could also be associated with genetics. Taken together, it is clear that BAV is not a simple Mendelian trait but an accumulation of complex traits (Ellison et al., 2007; McBride et al., 2008) and is indeed heritable; therefore, we cannot treat BAV as a homogenous patient population.

### Valvulopathy

The different phenotypes of valvulopathy in BAV patients also reflect the heterogeneity of this patient population. In all age groups, BAV underlies the majority of cases of aortic valve disease (Martin et al., 2007). The most common complication of BAV is valvular stenosis followed by valvular insufficiency (Pachulski and Chan, 1993). In patients that present with valvular dysfunction earlier in life (<50 years old), AI is more common; however, later in life (>50 years old) aortic stenosis (AS) is more prevalent (Pachulski and Chan, 1993). Studies have suggested a correlation between BAV phenotype and the valvular complications that develop. The overall evidence suggests that R-N BAV phenotypes have the greatest incidence of AS in both children and adults (Fernandes et al., 2004, 2007; Huang and Le Tan, 2014; Adamo and Braverman, 2015). In pediatric patients, those with the R-N BAV phenotype were more likely to have AI progression (Fernandes et al., 2004). Patients with R-N BAV have a high prevalence of AI and AS, therefore, the AI could be due to the high incidence of AS, while patients with R-L and N-L BAV phenotypes could have a high incidence of AI due to a separate mechanism (Fernandes et al., 2004, 2007). Research shows that BAV insufficiency and BAV stenosis have noticeably different characteristics. Hahn et al. (1992) demonstrated that dilation of the aortic annulus and entire aortic root is associated with AI.

### Aortic dilation/aneurysm

Compared to the normal population, there is a significantly higher rate of dilation of the proximal aorta in patients with BAV (Della Corte et al., 2007). Della Corte et al. (2007) found that aortic dilation [an aortic ratio (measured diameter/expected diameter) >1.1] was present in 83.2% of patients with BAVs, 79% mid-ascending dilation and 58% root dilation in adults. Aortic aneurysm commonly involves aortic root, ascending aorta, and aortic arch in clusters (Fazel et al., 2008). Researchers suggest two theories for the cause of aneurysms in patients with BAV: the hemodynamic theory and the aortopathy theory.

While the hemodynamic theory was the first explanation for BAV-associated aortic aneurysm, the genetic/aortopathy theory has become increasingly popular. Upon pairing patients with TAVs and BAVs based on sex and degree of AI, stenosis, or combined aortic valve disease, the patients with BAV were considerably younger due to the earlier onset of valvular disease and had significant aortic dilation at all levels compared to the matched patients with TAVs (Keane et al., 2000). Matching patients with TAVs and BAVs with similar degrees of valvular abnormalities reduces the effect of valvular lesions on hemodynamics and more accurately assesses the BAV (Keane et al., 2000). Hahn et al. (1992) established that aortic dilation is common in patients with BAVs even when the hemodynamic function of the valve is normal, providing support for the genetic/aortopathy theory.

The exact molecular and cellular pathways involved in BAV aortopathy remain unknown. However, MMP-2 (matrix metalloproteinase-2) has been identified as a key molecular modulator and a circulation biomarker of aortic dilation in patients with BAV. An increase in MMPs, enzymes that process or degrade the extracellular matrix, is associated with the development of aortic aneurysms (Ikonomidis et al., 2007). A study of patients with TAA comparing patients with BAVs and patients with TAVs, found that MMP-2 was increased by 34% in patients with BAVs (Ikonomidis et al., 2007). Therefore, an increase in collagen turnover and a decrease in collagen cross-linking may be a factor in the formation of aneurysms in patients with BAVs (Broberg and Therrien, 2015).

The pathological hallmark of TAA is medial degeneration; therefore, smooth muscle cells (SMCs; key component of the medial layer) function in this pathology (Milewicz et al., 2008). Mutations in SMC-specific contractile proteins could contribute to familial TAA (Milewicz et al., 2008). Various mutations in both MYH11 and ACT2A disrupt SMC contractile function causing TAA (Milewicz et al., 2008). Through the use of iPSCs modeling the BAV/TAA, we found that the defective differentiation of SMCs from neural crest stem cells, modeling the root, and ascending aortic aneurysm, manifested as decreased expression of *MYH11* and contractile function of SMCs (Jiao et al., 2016). On the other hand, the SMCs differentiated from paraxial mesoderm, modeling the descending thoracic aorta, have normal expression of contractile protein, including *MYH11* and contractile function (Jiao et al., 2016). This finding in our cellular model is consistent with clinical observation that the TAA in patients with BAV involves the root and ascending aorta but spares the descending thoracic aorta, indicating the aortopathy in BAV/TAA.

While aortopathy is important in TAA formation in patients with BAVs, the hemodynamic theory cannot be ignored. Similar to the valvulopathy, BAV subtype exhibits a correlation to the location of aortic dilation. Hope et al. (2010) found left-posterior flow jets and left-handed nested helical flow only in R-N BAVs, more distally directed flow derangement with tubular ascending and arch dilation. Hope et al. (2010) found right-anterior flow jets and right-handed nested helical flow, only seen in patients with R-L BAVs, were related to proximal flow derangement and dilation of convex side of the ascending aorta. Due to the different flow patterns produced by R-L and R-N BAVs, R-L BAVs more often cause dilation of ascending convex side while R-N BAVs cause ascending and arch dilation. BAVs compared to TAVs and different subtypes of BAV alter the hemodynamic blood flow through the valve, diversely impacting the aorta, leading to different locations of dilation. While the fusion subtypes contribute to flow patterns, so do valvular dysfunctions, such as AI and AS. Most frequently, the hemodynamic change in patients with BAV affects the ascending aorta, but not the aortic root (Tadros et al., 2009). Aortic regurgitation yields higher stroke volumes which causes higher wall tension in the ascending aorta while AS creates a high-velocity jet that increases shear stress on the ascending aorta (Tadros et al., 2009). Yet, aortic root aneurysms are still frequently seen in BAV patients (Schaefer et al., 2008). This fact supports the idea that aortopathy is the key factor of TAA in patients with BAV, and that patients with root aneurysms may be due to more severe aortopathy and less to hemodynamics. Also, patients with BAV and isolated dilation of the aortic root tend to be younger, are more likely male, and have AI (Tadros et al., 2009; Girdauskas et al., 2010, 2012; Detaint et al., 2014).

Taken together, BAV is a heterogeneous disease. Different gene mutations may cause different subtypes of BAV and exhibit different severity of aortopathy, which result in aortic aneurysm in different parts of the aorta (root, ascending aorta or arch) and at different ages with the contribution of hemodynamic change due to BAV. An asymptomatic ascending aortic aneurysm in a 60-year-old BAV patient with Sievers type 1, L-R fusion, and AS is different from an asymptomatic aortic root aneurysm in a 30-year-old BAV patient with Sievers type 0 and AI. The criteria of surgical repair of the aneurysm in these two patients should be different.

### Aortic dissection

Aortic dissection occurs 5–10 times more often among patients with BAVs than those with TAVs (Braverman, 2011), rendering it a potentially lethal disease. In patients with BAVs followed prospectively, aortic dissection has been an infrequent event; however, in patients with BAVs aortic dissection occurs an average of one decade earlier than patients with TAVs (Braverman, 2011).

A study of 56 patients with pure AI, who had an isolated AVR found that the subset of patients with a root aneurysm and AI appears to be different than the hemodynamically-triggered aortopathy seen in patients with BAV stenosis and asymmetric dilation of the tubular ascending aorta (Girdauskas et al., 2015a). This root phenotype (root aneurysm and AI) of BAV has been linked to more of a genetic/aortopathy cause, and occurs earlier in life and independent of hemodynamic factors (Della Corte et al., 2007; Girdauskas et al., 2015a). The incidence of adverse events was substantially higher in patients with the root phenotype of BAV as opposed to those with BAV/ascending aortic aneurysm and AS (Girdauskas et al., 2012). Girdauskas et al. found that patients with BAV-AI had a 10-fold higher risk of post-AVR aortic dissection when compared to patients with BAV-AS (Girdauskas et al., 2015b). A study by Wang et al. (2016) found that patients with BAV-AI had a higher prevalence of R-L fusion phenotypes and wider aortic roots than patients with BAV-AS. Girdauskas et al. (2015a) found that 34% of participants with BAV and root phenotype suffered aortic complications after AVR and only 50% of patients in the study were unburdened by aortic complications 15 years post-AVR. Girdauskas et al. reported that 3/56 patients with root phenotype expired due to type A dissection, while 0/153 patients with stenosis and ascending aortic dilation suffered a Type A aortic dissection (Girdauskas et al., 2012, 2015a). Both Girdauskas et al. (2012) and Wang et al. (2016) found that patients with BAV-AI and root aneurysm are closer to Marfan syndrome (MFS) pathology and have a higher risk of aortic dissection and rupture. When comparing patients with BAV and MFS, Itagaki et al. (2015) found that the risk of aortic complications after AVR was 10-times higher for patients with MFS than for patients with BAV, but patients with BAV were at a significantly greater risk than patients with acquired disease of a tricuspid aortic valve. However, this study looks at all patients with BAV together, despite the proven heterogeneity of the BAV population. Therefore, though it seems that BAV finds itself in the middle of the high risk of patients with MFS and the low risk of patients with acquired valve disease of a TAV, by separating out the distinct populations of BAV, we believe those with the root phenotype (root dilation and AI) will be closer to those of MFS while those with AS and ascending dilation will be closer to those of the acquired valve disease of the TAV. Therefore, it might be reasonable to separate patients with BAV into groups to decide treatment; potentially be more aggressive when treating patients with BAV—with root phenotype (AI and root aneurysm).

We propose that when evaluating patients, patients can be classified into two separate groups; Cluster A—root phenotype (root aneurysm and AI) and Cluster B—no root involvement (no root aneurysm or AI). While the percentage of patients with Cluster A aortopathy is often less than those with Cluster B, Cluster A aortopathy is associated with a faster diametric growth rate of the ascending aorta (Della Corte et al., 2013) and high prevalence of aortic events after AVR (Girdauskas et al., 2015a). These findings suggest that this Cluster A aortopathy could be a “malignant” form of BAV that could be genetically linked; while Cluster B could be the benign form. Because patients with Cluster A aortopathy have an increased probability of life-threatening aortic events, such as aortic dissection and rupture, they could fit under the more aggressive guidelines for patients with connective tissue disorders, such as MFS (Girdauskas et al., 2015a), with recommendation of surgical resection of a root aneurysm at a diameter of 5 cm, as did Michelena et al. (2015), if asymptomatic and 4.5 cm as a concomitant surgery. Cluster B are more affected by the hemodynamics, can be treated as TAV/TAA with surgical resection at 5.5 cm if asymptomatic. Regarding those two hypothetical cases above, we would recommend a conservative approach for the first patient (60-year-old with BAV and ascending aneurysm/AS) with surgical resection when the aneurysm reaches 5.5 cm, as recommended by the current AHA/ACC guidelines; an aggressive approach for the second patient (30-year-old with aortic root aneurysm and AI) with surgical resection when the aneurysm reach 5 cm as the recommendations of current AHA/ACC guidelines for the MFS.

## Discussion

**Figure 1:**
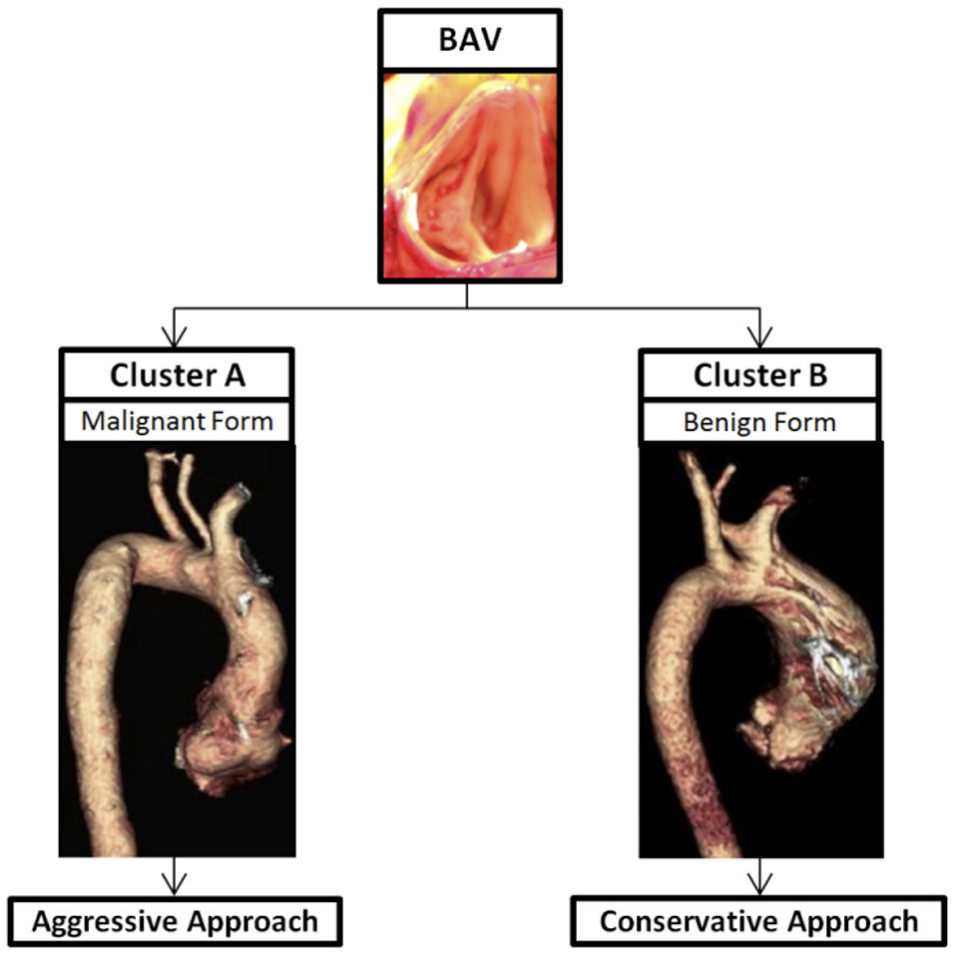
Thoracic aortic aneurysm in BAV, a heterogeneous disease, can be divided into two clusters based on the aortic root involvement. Cluster A is BAV/TAA with aortic root aneurysm—a malignant form and should be treated more aggressively. Cluster B is BAV/TAA without aortic root dilation—a benign form and should be treated conservatively.

Patients with BAV present with different genetics, BAV subtypes, valvular complications, and areas of aortic dilation; therefore, instead of treating BAV as a homogenous disease, it should be treated based on the different subtypes and associated valvulopathy/aortopathy. Cluster A BAV (malignant form)—root phenotype with aortic root aneurysm and AI should be treated more aggressively with surgical resection at 5 cm for asymptomatic patients, as for patients with MFS, while Cluster B BAV (benign form) without aortic root aneurysm could be treated less aggressively as for patients with a tri-leaflet aortic valve with surgical resection at 5.5 cm for asymptomatic patients (Figure 1). Concomitant elective surgery of the aorta should be considered in both Cluster A and B when undergoing clinically indicated AVR and the aorta measures ≥ 4.5 cm. A study by Michelena et al. (2011) found that patients with BAV and an aortic aneurysm >4.5 cm were eight times more likely to undergo an aortic dissection. Schneider et al. (2017) found that a concomitant aortic root remodeling procedure by resecting aortic sinuses in patients with BAV undergoing an aortic valve repair when the patient's root exceeded 4.2–4.5 cm has good 10–15 year results. Therefore, when a 30-year old BAV patient needs an operation for severe AI and congestive heart failure, this patient should undergo aortic root replacement if a root diameter ≥ 4.5 cm instead of an isolated AVR. While the addition of an aortic root procedure to an AVR increases technical complexity as well as cardio-pulmonary bypass time, Kim et al. found that the procedure addition did not result in an elevated operative risk, a prolonged postoperative course, or an increased blood transfusion (Kim et al., 2012). However, if surgeons are not familiar with the aortic root procedures, they should refer the case to a high-volume center of performing aortic root procedures to achieve the best outcome.

## Author contributions

EN and BY substantially contributed to the conception and design of the work, the acquisition, analysis of data for the work; drafted and revised the work critically for important intellectual content; approved the final version to be published; and agree to be accountable for all aspects of the work ensuring that questions related to the accuracy or integrity of any part of the work were appropriately investigated and resolved.

### Conflict of interest statement

The authors declare that the research was conducted in the absence of any commercial or financial relationships that could be construed as a potential conflict of interest.

## Abbreviations

AI: aortic insufficiency
AS: aortic stenosis
AVR: aortic valve replacement
BAV: bicuspid aortic valve
TAA: thoracic aortic aneurysm
TAV: tricuspid aortic valve
MFS: Marfan syndrome.
